# Ameliorating Effect of Ginseng on Epididymo-Orchitis Inducing Alterations in Sperm Quality and Spermatogenic Cells Apoptosis following Infection by Uropathogenic *Escherichia coli* in Rats 

**DOI:** 10.22074/cellj.2016.4573

**Published:** 2016-08-24

**Authors:** Mehdi Eskandari, Soghra Jani, Mahsa Kazemi, Habib Zeighami, Alireza Yazdinezhad, Sahar Mazloomi, Saeed Shokri

**Affiliations:** 1 Department of Physiology, Faculty of Medicine, Zanjan University of Medical Sciences, Zanjan, Iran; 2 Department of Anatomical Sciences, Faculty of Medicine, Shahid Beheshti University of Medical Sciences, Tehran, Iran; 3 Department of Microbiology, Faculty of Medicine, Zanjan University of Medical Sciences, Zanjan, Iran; 4 Department of Pharmacognosy, Faculty of Pharmacy, Zanjan University of Medical Sciences, Zanjan, Iran; 5 Metabolic Diseases Research Center, Faculty of Medicine, Zanjan University of Medical Sciences, Zanjan, Iran; 6 Department of Anatomical Sciences, Faculty of Medicine, Zanjan University of Medical Sciences, Zanjan, Iran

**Keywords:** Ginseng, Uropathogenic * Escherichia coli*, Sperm, Rat, Testis

## Abstract

**Objective:**

Epididymo-orchitis (EO) potentially results in reduced fertility in up to 60% of affected patients. The anti-inflammatory effects of Korean red ginseng (KRG) and its ability to act as an immunoenhancer in parallel with the beneficial effects of this ancient herbal medicine on the reproductive systems of animals and humans led us to evaluate its protective effects against acute EO.

**Materials and Methods:**

This animal experimental study was conducted in the Department of Anatomical Sciences, Faculty of Medicine, Zanjan University of Medical Sciences (ZUMS), Zanjan, Iran during 2013-2015. We divided 50 Wistar rats into five following groups (n=10 per group): i. Control-intact animals, ii. Vehicle-phosphate buffered saline (PBS) injection into the vas deferens, iii. KRG-an intraperitoneal (IP) injection of KRG, iv. EO-an injection of uropathogenic * Escherichia coli* (UPEC) strain M39 into the vas defer- ens, and v. EO/ KRG-injections of both UPEC strain M39 and KRG. The treatment lasted seven days. We then evaluated sperm parameters, number of germ cell layers, Johnson’s criteria, germ cell apoptosis, body weight and relative sex organs weight.

**Results:**

Acute EO increased the relative weight of prostate and seminal vesicles (P≤0.05). It also reduced sperm quality such as total motility, sperm concentration (P≤0.01), and the percentage of normal sperm (P≤0.001). Moreover, acute EO decreased Miller’s (P≤0.05) and Johnsen’s scores and increased apoptotic indexes of spermatogenic cells (P≤0.001). KRG treatment decreased prostate weight gain (P≤0.05) and improved the percentage of sperm with normal morphology, total motility (P≤0.01), and progressive motility (P≤0.05). The apoptotic indexes of spermatogenic cells reduced (P≤0.001), whereas both Johnsen’s (P≤0.01) and Miller’s criteria increased in the KRG-treated EO testis (P≤0.05).

**Conclusion:**

Consequently, KRG ameliorated the devastating effects of EO on the sperm retrieved from either epididymis or testicle in rats.

## Introduction

An urogenital tract infection (UTI) in men is caused by uropathogenic * Escherichia coli* (UPEC) that is also known as one of the most common causes of epididymo-orchitis (EO) (1,2). EO caused by either sexual transited pathogen (3) or UTI (4) negatively affects fertility (2,5). Acute epididymitis can result in azoospermia by bilateral ductal obstruction or destruction of the seminiferous tubules (6). Moreover, it can lead to recurrent oligoasthenoteratozoospermia through defective spermatogenesis of inflamed testis in up to 60% of patients (7,8). Although antibiotic treatments are considered as a standard therapy in acute bacterial EO (9), successful elimination of invading pathogens does not necessarily mean that fertility can be fully reversible (8,10,11). Apart from the possibility that viable bacterium inside the epididymis or prostate tissue may sustain chronic inflammation; there is a second hypothesis of a pathogenic pathway for persistent chronic inflammation of the epididymis as a nonbacterial process after eradication of the causative agent. Furthermore, even after obliteration of the invading pathogen by the host immune system within the testis, impairment of spermatogenesis remains irreversible (12). 

Ginseng [Korean red ginseng (KRG)] is used as a medicine in East Asia countries. Ginsenosides (triterpene saponins) are the major active ingredients of ginseng (5,13). Ginseng is a powerful antioxidant with an extensive range of functions (14,17) including induction of spermatogenesis and activation of either glial cell line-derived neurotrophic factor (GDNF) (18) or cyclic adenosine 3´, 5´-monophosphate (cAMP)-responsive element modulator (CREM) (19) in rat testes. Anti-aging effects of ginseng on testes function have also been documented (20). It is well established that ginseng exhibits a therapeutic capacity against toxic effects of environmental contaminants (21,22) and chemotherapeutic drugs by protecting testicular function and improving sperm quality in the animal (23,24). Different studies conducted on animal models have found that ginsenosides, major active ingredients of ginseng, increase luteinizing hormone (LH) secretion (25), enhance sperm motility, as well as promote sperm progression (26), capacitation and acrosome reaction (27). Clinically, KRG enhances sexual functions (28), such as libido and mating performance (29,30). Clinical trials have also confirmed that ginseng extract facilitates erectile dysfunction (ED) (31,32), improves sperm progressive motility, as well as increases the levels of spermatozoa, plasma total, free testosterone, dihydrotestosterone, follicle-stimulating hormone (FSH), and LH (33,34) in fertile and asthenozoospermic men (24,35). 

In general, common inflammatory reactions in epididymis and/or testis lead to significant damage to testicular function and loss of spermatogenesis (36). The testicular immune response is, therefore, rendered ineffective against UPEC (37). The anti-inflammatory effects of KRG and its ability to act as an immune-enhancer (15,38,39) in parallel with the mentioned beneficial effects of this ancient herbal medicine on the reproductive systems of animals and humans led us to evaluate its protective effects against acute EO. 

## Materials and Methods

This animal experimental study was conducted in the Department of Anatomical Sciences, Faculty of Medicine, Zanjan University of Medical Sciences (ZUMS), Zanjan, Iran, during 2013-2015. 

### Animals

Healthy adult male Wistar rats (250-280 g) were purchased from Pasture Institute, Tehran, Iran. Animals were kept on a 12-hour light-dark cycle at 23 ± 2˚C and fed with standard pellets and water ad libitum. All animal experiments were conducted in accordance with national guidelines and protocols, approved by the Institutional Animal Ethics Committee (IAEC No.03/028/07). All experimental protocols were approved by the Ethics Committee of ZUMS. 

### Treatments

Following one-week acclimatization, we divided 50 adult rats into following five groups (n=10 per group): i. Control-intact animals receiving no treatment or surgery, ii. Vehicle-animals undergoing surgery and receiving an injection of normal saline, iii. KRGanimals undergoing no surgery and receiving an intraperitoneal (IP) injection of KRG (15 mg/kg/daily) for seven days, iv. EO-animals undergoing surgery and receiving an injection of UPEC strain M39, and v. EO/ KRG-animals undergoing surgery and receiving injections of both UPEC strain M39 and KRG (15 mg/kg/daily) for seven days. The dosage was based on the Office of Monopoly, Republic of Korea (ROK)’s prescription, and the method of Hess et al. (40) sub-acute toxicity study. It has showed that IP injection of Ginseng 15 mg/kg daily has no toxic effects on different organs of rats. Seven-day therapy was selected based on the study of Lu (41). 

### Proliferation of the bacterial strain

UPEC strain M39 was isolated from the urine of a male child who was less than 5 years old, experienced UTI and was resident of Tehran, Iran. 

According to a standard laboratory protocol, the hemolytic (Hly) strains were isolated, cultured and proliferated overnight on Columbia blood agar plates (Merck, Germany). Chloramphenicol (Sigma, USA, 20 μg/ml) was then added as a supplement to the lysogeny broth (LB, Merck, Germany) medium. LB medium was applied to culture the fresh cells in a shaking incubator at 37˚C, for the early exponential phase of growth [optical density (OD) 600=0.5~1.0]. The concentration of viable bacteria was calculated by the standard growth curves. The bacteria (2×10^9^ cfu) were then centri- fuged at 4500 g for 8 minutes at room temperature. TThe pellet was washed with phosphate buffered saline (PBS, Merck, Germany) and resuspended in 10 ml saline. For *in vivo* experiments, the bacterial suspension was diluted by sterile saline to 5×10^5^cfu in 50 μl (OD600=0.06). A pilot study was performed with different concentrations (OD600=0.2, 0.1, 0.08, 0.06, and 0.04) of bacterial suspensions in order to determine the ideal concentration that could generate a rat model of EO. The ideal concentration under our laboratory conditions was OD600=0.06 (42). 

### Bacterial-induced experimental epididymo-orchitis

Briefly, male rats were anesthetized by IP injection of ketamine (45 mg ⁄kg) and xylazine (35 mg ⁄kg) mixture (Ciron Drugs & Pharmaceuticals Pvt Ltd, India). The testis, epididymis and vas deferens were exposed by a scrotal incision. A total of 50 μl of UPEC strain M39 suspension (approximately 5×10^5^ bacteria) was injected into the vas deferens of each side by a 30-gauge needle. The vas deferens from each testicle was clamped at the injection site to prevent spread of the infection. Vas deferens dilation with a transparent cauda epididymis that showed no fluid leakage from the injection site was assumed as a successful injection procedure. AAfter surgery, the animals were kept under standard conditions and in individual cages until sacrificed with an overdose of isoflurane (Gurgaon, India) (42,43). 

### Uropathogenic * Escherichia coli* detection in the testis

Testicles from either infected or uninfected rats were homogenized in 10 ml sterile PBS with a sterile glass potter. A total of 100 μl of each homogenized testis was streaked on an agar plate and incubated at 37˚C overnight. Bacterial colonies were checked the next morning under translucent light (42). 

### Ginseng preparation

A batch of KRG was purchased from an herbal drugstore, Tehran, Iran, and ground into a dried powder. The prepared powder (30 g) was mixed with 600 ml ethanol (Merck, Germany, 50%). The herbal mixture was boiled continuously to reach half of the original volume, after which the suspension was centrifuged at 10000 g for 30 minutes. The supernatant was collected and dried in a SpeedVac System (Freeze Dryer alpha 1-2/ LD plus, Martin Christ, Germany). The dried extract was mixed with sterile PBS to make a stock of 100 mg extract/ml. A 0.2-µ filter (Millipore, USA) was used for sterilizing the extract solution (44,45). 

### Body weight and reproductive organs weight

Animals were anesthetized with an IP injection of a ketamine (45 mg ⁄kg) and xylazine (35 mg ⁄kg) mixture. The final body weights were measured. One side of the testicle was randomly dissected and weighed. After homogenizing the dissected testicle, 100 mg of the homogenized tissue was transferred into 1 ml PBS and stored at -20˚C. This tissue was used to perform an enzyme-linked immunosorbent assay (ELISA, Thermo Fisher Scientific Inc., USA) to determine the tumor necrosis factor-alpha (TNF-α) concentration. The other testicle and remainder of the sex organs (epididymis, seminal vesicles and ventral prostate) were dissected out and weighed. The body weight gain during the experiment was calculated by subtracting the final body weight from the initial weight. The relative weights of the reproductive organs were also measured. Testicular tissues were fixed in 10% buffered formaldehyde solution and embedded in paraffin wax. The sections, 5 µm each, were cut and prepared for staining with a terminal deoxynucleotidyl transferase dUTP nick end labeling (TUNEL) assay kit (Roche, Germany) for detecting germ cells apoptosis. 

### Sperm characteristics

Epididymal sperm were collected by dissecting the caudal part of the epididymis on both sides. Sperm were separated from epididymal tubules by chopping the caudal part of the epididymis in 5 ml of Hams F10 solution (Sigma-Aldrich, USA). The solution was incubated for 5 minutes at 37˚C. After pipetting, one drop of sperm suspension was placed on a microscope slide and cover slipped. At least ten microscopic fields were observed at ×400 magnification, while in situ motility, progressive motility and immotile sperm were expressed as a percentage of the total sperm count according to Shokri et al. (46). After evaluating the sperm motility, a prepared slide was evaluated for abnormal sperm in at least ten fields at ×400 magnification. Sperm that lacked tails, as well as those with morphologically abnormal heads and tails were counted. The abnormal sperm was expressed as a percentage of total counted sperm. The epididymal sperm counts were obtained using the method described by Shokri et al. (46). Briefly, the suspension was diluted with saline that contained 0.5% formalin, after which it was placed on an erythrocytometer (Neubauer type, Hausser Scientific, USA) and examined under a light microscope (Olympus, Japan) to determine the sperm count (47). 

### Evaluation of spermatogenesis by Johnsen’s and Miller’s scores

We categorized spermatogenesis by measuring the number of germinal cell layers and Johnsen’s score (as a score of 1-10) in the testes. Briefly, the number of germinal epithelial layers was counted in ten seminiferous tubules as described by Miller et al. (47). The scores were according to the presence or absence of the main cell types arranged in the order of maturity. 

### Germ cell apoptosis by TUNEL kit

Germ cell apoptosis was evaluated by terminal deoxynucleotidyl transferase (TdT) enzyme mediated by TUNEL assay kit (Roche, Germany) according to the manufacturer’s instruction. Briefly, the 5-μm thick paraffin-embedded sections were microwave-pretreated in 10 mM citrate buffer (pH=6.0, Merck, Germany) for 10 minutes. Sections were incubated with blocking solution (3% H_2_O_2_ in methanol, Merck, Germany) for 10 minutes and then washed with PBS. The specimens were incubated with TUNEL reaction mixture (TdT and nucleotide mixtures in reaction buffer) at 37˚C for 60 minutes. Finally, the slides were stained with converter- peroxidase (POD, Roche, Germany, anti-fluorescein antibody, Fab fragment from sheep, conjugated with horse-radish POD) for 30 minutes. At the last stage, the 3, 3-diaminobenzidine (DAB) substrate (Roche, Germany) was applied to develop a brownish stain in fragmented nuclear chromatin of the apoptotic cells. Number of apoptotic cells was counted by a light microscope (Olympus, Japan) at ×400 magnification. From each testis, a minimum of 10 sections were selected for quantification. And, in each section, 10 randomly selected seminiferous tubules were quantified. Therefore, apoptotic cells were counted for at least 100 seminiferous tubules in each tissue. Brownish nuclear staining was considered as a positive apoptotic cells. Two following apoptotic indices were used for evaluation: i. Apoptotic index-1 (AI-1) defined as the number of TUNEL-positive apoptotic cells per 100 tubules and ii. . Apoptotic index-2 (AI-2) defined as the number of tubules containing TUNEL-positive apoptotic cells per 100 tubules (47).

### Immunoassay of tumor necrosis factor alpha

A commercial rat TNF-α ELISA kit (CUSABIO, China) was used to quantify TNF-content in the testis tissue. Samples were measured in duplicate. The minimum detectable concentration of rat TNF-α was <1.56 pg/ml. All procedures were performed according to the manufacturer’s instructions.

### Statistical analysis

Data were expressed as mean ± SE. The oneway analysis of variance (ANOVA) test was applied to clarify significant differences among groups. When a significant effect was found, the Tukey’s test was performed. All analyses were performed using the Statistical Package for the Social Sciences (SPSS, SPSS Inc., USA) version 16. The statistical significance level was set at P≤0.05. 

## Results

### Invasion and localization of Uropathogenic * Escherichia coli* inside testis

**Fig.1:**
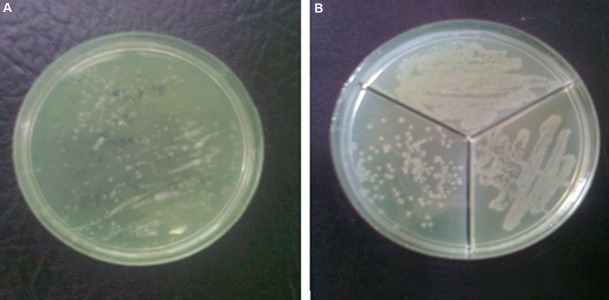
UPEC detection in the testis using an agar plate. A. Testicular homogenates from sterile PBS injected and B. UPEC infected rats were streaked on agar plates without antibiotics and kept at 37˚C overnight. Colonies were counted under translucent light. In plate B, the up- per third of the plate was related to the bacteria UPEC strain C69. The lower third of the same plate in the right side was related to the bacteria UPEC strain M39, and lower third of the plate in the left side is related to the bacteria UPEC strain C69. In this study, we selected UPEC strain M39. UPEC; Uropathogenic * Escherichia coli* and PBS; Phosphate buffered saline.

Initially, all infected animals developed clinical symptoms of acute epididymitis in the scrotum. Inflammation ranged from a mildly edematous scrotum to severe enlargement and erythema of the scrotal wall. Figure 1 shows the presence and localization of UPEC in the testis. Testicular homogenates with sterile PBS injected (Plate A) and UPEC infected rats (Plate B) were streaked on agar plates (without antibiotic) and kept at 37˚C overnight. Colonies were counted under translucent light. In plate B, the upper third of the plate was related to the UPEC strain C69. The lower third of the same plate in the right side was related to the UPEC strain M39 and lower third of the plate in the left side was related to the bacteria UPEC strain C69. In this study, we selected UPEC strain M39 due to the strong intensity. 

### Body weight and sex organs weight 

The relative weight of sex organs were compared among groups (Table 1). We observed no significant difference regarding to the relative weights of sex organs in the KRG and vehicle groups as compared to the control group. There was a significant increase in the relative weight of the prostate [0.63 ± 0.21 (EO group) vs. 0.17 ± 0.02 (control group), P≤0.05], left seminal vesicle [0.15 ± 0.03 (EO group) vs. 0.06 ± 0.0 (control group), P≤0.01] and right seminal vesicle [0.13 ± 0.0 (EO group) vs. 0.06 ± 0.01 (control group), P≤0.05] of EO animals as compared to the control group. Fluctuations in body weight were not significantly different between the control group with both vehicle and KRG groups. EO induction caused a significant reduction in body weight that was defined as a negative weight loss in EO group (36.66 ± 19.22 g) as compared to a positive weight gain of control group (10 ± 2.88 g, P≤0.01). 

### Sperm parameters

The effect of ginseng treatment on the sperm parameters are shown in Table 2. Although the vehicle group showed no significant difference in the percentage of normal sperm, there was a significant reduction (2.00 ± 0.35) in sperm count as compared to the control group (3.29 ± 0.00, P≤0.05). We observed no significant differences in terms of the sperm count and percentage of normal sperm between the KRG and control groups (Table 2). Interestingly, in EO group, EO resulted in a significant reduction in sperm count (0.43 ± 0.11) and the percentage of normal sperm (53.27 ± 5.97) as compared to the relative values (2.00 ± 0.35, P≤0.01 and 90.18 ± 1.42, P≤0.01, respectively) of vehicle group. Although ginseng administration did not prevent reduced sperm count in EO animals, it insignificantly increased the sperm count from 0.43 ± 0.11 in the EO group to 1.78 ± 0.33 in the EO/KRG group (P≤0.01). Ginseng significantly increased the percentage of sperm with normal morphology in the EO/KRG group (79.93 ± 2.90) as compared to the EO group (53.27 ± 5.97, P≤0.001). According to Table 2, there was no significant difference regarding the percentage of sperm progressive motility between the vehicle and control animals. On the other hand, there was a significant reduction in the percentage of total motility in the vehicle (58.26 ± 3.87) group as compared to the control group (77.66 ± 6.40, P≤0.05). 

Interestingly, ginseng administration to intact animals significantly increased the percentage of sperm progressive motility (36.98 ± 6.52) as compared to the control group (16.20 ± 1.23, P≤0.05). There was no significant fluctuation in the percentage of total motility in the KRG group. The percentage of total motility parameter was significantly reduced in the EO group (24.19 ± 9.54) as compared to the vehicle group (58.14 ± 3.87, P≤0.01). Ginseng administration to the EO animals caused a significant increase in the percentage of total motility parameter in the EO-KRG group (62.77 ± 4.36) as compared to the EO group (24.19 ± 9.54, P≤0.01). The same pattern of increase was observed in the percentage of sperm progressive motility of EO/KRG group (27.77 ± 1.53) as compared to the EO group (7.67 ± 2.23, P≤0.05). 

### Bacterial agglutination of sperm

**Fig.2:**
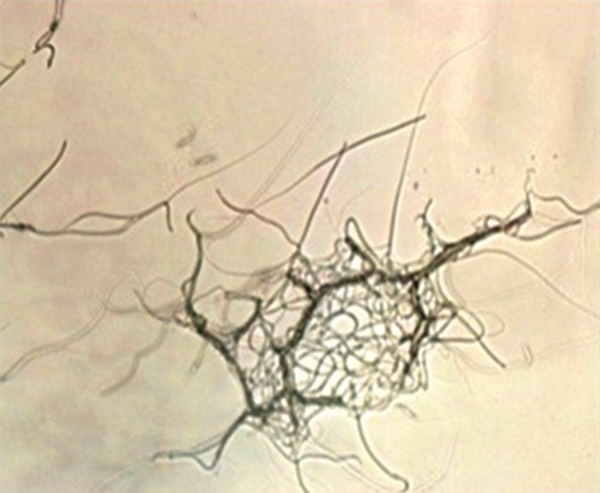
Multiple adhesions of * Escherichia coli* to spermatozoa (×40 magnification).

Figure 2 shows that *E-coli* caused agglutination by attaching to human spermatozoa.

### Germ cells apoptosis

**Fig.3:**
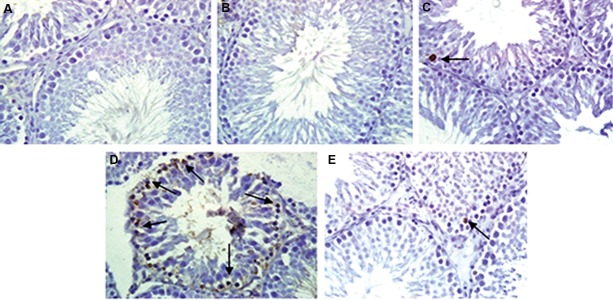
Representative analysis of germ cell apoptosis (TUNEL positive cells) in cross-sectioned testis of experimental groups. A. Control, B. Vehicle, C. KRG, D. EO/KRG, and E. EO groups (×40 magnification). Arrows indicate TUNEL positive cells. TUNEL; Terminal deoxynucleotidyl transferase dUTP nick end labeling, KRG; Korean red ginseng, and EO; Epididymo-orchitis.

**Fig.4:**
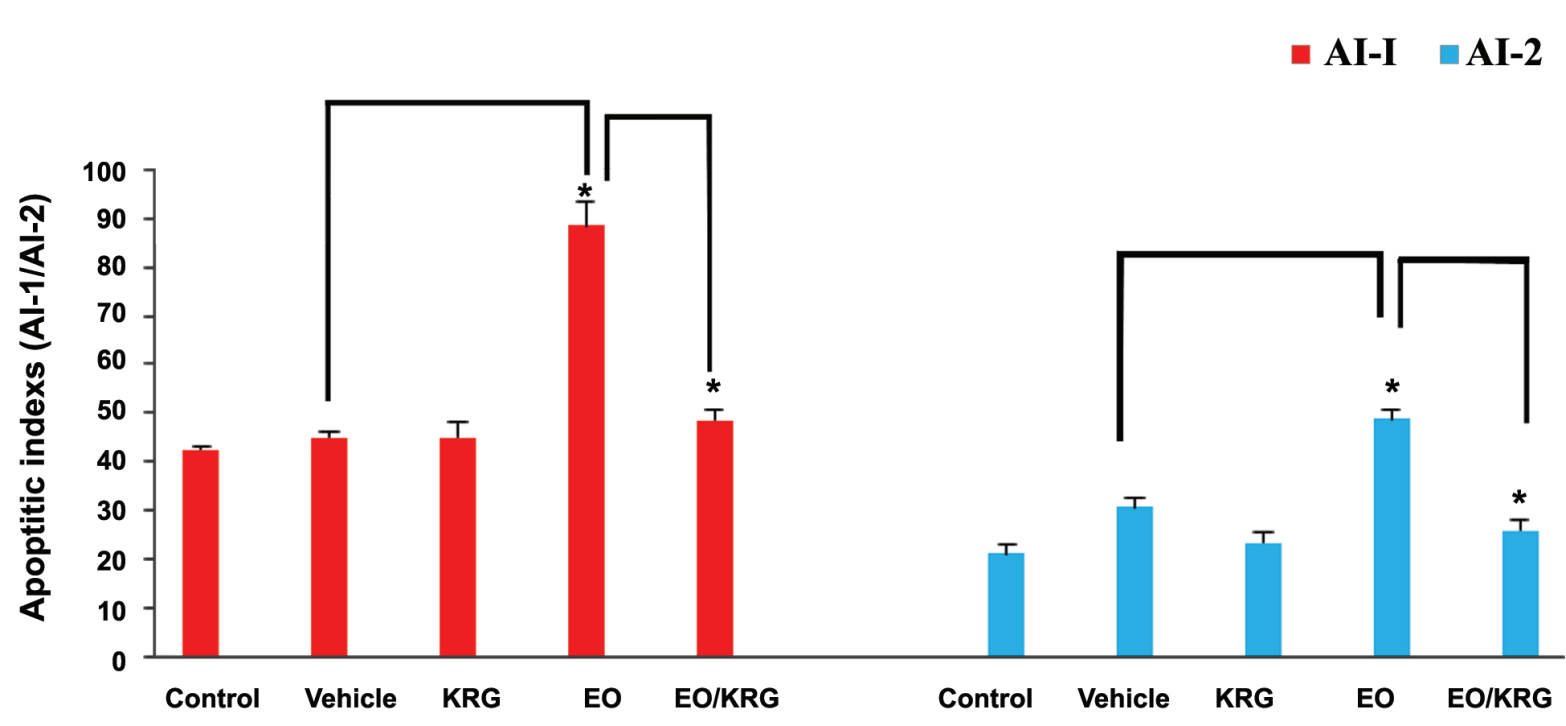
The effect of EO and ginseng treatment on the rat testicular germ cell apoptosis. Histogram represents the number of apoptotic cells per 100 tubules (AI-1) and the number of tubules that have positive cells per 100 tubules (AI-2). *; P≤0.001, KRG; Korean red ginseng, EO; Epididymo-orchitis, AI-1; Apoptotic index-1, and AI-2; Apoptotic index-2.

Descriptive representation of TUNEL positive cells in the testis of experimental groups is shown in Figures 3 and 4. There were no significant differences in the AI-1 and AI-2 of both vehicle and KRG groups as compared to the control group. Ginseng treatment significantly decreased the number of TUNEL positive cells in the EO/KRG group as compared to the untreated infected animals [89.22 ± 3.71 (AI-1 in EO group) vs. 47.84 ± 0.96 (AI-1 in EO/KRG group), P≤0.001). AI-2, the number of TUNEL positive tubules, reduced significantly in the EO/KRG group (25.34 ± 0.46) as compared to the untreated EO animals (47.36 ± 1.11, P≤0.001). 

### Johnsen’s and Miller’s scores

According to Table 3, Johnsen’s score significantly decreased in the EO animals (6.95 ± 0.34, P≤0.001) as compared to the vehicle animals (9.3 ± 0.2). Concomitantly, Miller’s score showed that the average thickness of seminiferous layers in the EO group (3.04 ± 0.09) was significantly lower as compared to the vehicle group (P≤0.05). EO/KRG animals caused a significant increase in both Johnsen’s (P≤0.01) and Miller’s (P≤0.05) criteria. There were no significant differences in either the Miller’s or Johnsen’s criteria in the vehicle and KRG groups as compared to the control group. 

### Tumor necrosis factor alpha concentration

TNF-α concentration did not significantly fluctuate in the experimental groups (Table 4). 

**Table 1:** Effects of EO and ginseng treatment on body weight and sex organs weight


Weight	Relative right testis weight (%)	Relative left testis weight (%)	Relative right epididymis weight (%)	Relative left epididymis weight (%)	Relative right seminal vesicle weight (%)	Relative left seminal vesicle weight (%)	Relative ventral prostate weight (%)	Weight gain (g)
Groups								

Control	0.47 ± 0.02	0.48 ± 0.01	0.16 ± 0.0	0.20 ± 0.02	0.06 ± 0.01	0.06 ± 0.0	0.17 ± 0.02	10 ± 2.88
Vehicle	0.46 ± 0.00	0.45 ± 0.03	0.11 ± 0.01	0.11 ± 0.01	0.09 ± 0.0	0.09 ± 0.01	0.20 ± 0.01	6.66 ± 1.66
KRG	0.51 ± 0.01	0.51 ± 0.01	0.20 ± 0.01	0.19 ± 0.0	0.08 ± 0.01	0.07 ± 0.0	0.23 ± 0.0	-6.66 ± 1.66
EO	0.50 ± 0.01	0.52 ± 0.04	0.15 ± 0.03	0.13 ± 0.02	0.13 ± 0.01	0.15 ± 0.03 2	0.63 ± 0.21 1	-36.66 ± 19.22^2^
EO/KRG	0.49 ± 0.05	0.50 ± 0.04	0.13 ± 0.01	0.13 ± 0.01	0.14 ± 0.02	0.13 ± 0.03	0.26 ± 0.03^1^	-15 ± 2.88


Each value represents mean ± SE. The vehicle and KPG groups were compared to the control group. The EO group was compared to the vehicle group. The EO/KRG group was compared to the EO group. KRG; Korean red ginseng, EO; Epididymo-orchitis, ^1^; P≤0.05, and^2^; P≤0.01.

**Table 2:** Effect of EO and ginseng treatment on sperm count, percentage of normal sperm and motility parameters


Groups	Control	Vehicle	KRG	EO	EO/KRG
Parameters					

Sperm count (×10^6^/ml)	3.29 ± 0.00	2.00 ± 0.35^1^	3.68 ± 0.21	0.43 ± 0.11^2^	1.78 ± 0.33
Normal sperm (%)	88.76 ± 0.03	90.18 ± 1.42	89.12 ± 1.20	53.27 ± 5.97^3^	79.93 ± 2.90^3^
Progressive motility (%)	22.89 ± 2.23	13.68 ± 2.42	36.84 ± 3.95^1^	7.67 ± 2.23	27.77 ± 1.53^1^
Total motility (%)	77.66 ±6.40	58.24 ± 3.87^1^	73.51 ± 1.82	24.19 ± 9.54^2^	62.77 ± 4.36^2^


Each value represents mean ± SE. The vehicle and KPG groups were compared to the control group. The EO group was compared to the vehicle group. The EO/KRG group was compared to the EO group. KRG; Korean red ginseng, EO; Epididymo-orchitis, ^1^; P≤0.05, ^2^; P≤0.01 and ^3^; P≤0.001.

**Table 3:** Effect of EO and ginseng treatment according to Johnsen’s and Miller’ scores


	Johnsen’s scores	Number of germ cell layers

Control	8.87 ± 0.36	4.28 ± 0.23
vehicle	9.3 ± 0.20	4.13 ± 0.20
KRG	9.3 ± 0.14	4.15 ± 0.32
EO	6.90 ± 0.34^3^	2.84 ± 0.35^1^
EO/KRG	8.44 ± 0.37^2^	4.06 ± 0.15^1^


Each value represents mean ± SE. The vehicle and KRG groups were compared to the control group. The EO group was compared to the vehicle group. The EO/KRG group was compared to the EO group. KRG; Korean red ginseng, EO; Epididymo-orchitis, ^1^; P≤0.05, ^2^; P≤0.01, and ^3^; P≤0.001.

**Table 4:** Effect of EO and ginseng treatment on TNF-α concentration


	Control	Vehicle	KRG	EO	EO/KPG

TNF-α concentration (pg/mL)	3.40 ± 1.05	2.77 ± 0.93	5.52 ± 0.93	4.83 ± 1.24	5.16 ± 0.98


Each value represents mean ± SE. TNF-α; Tumor necrosis factor alpha, EO; Epididymo-orchitis, and KRG; Korean red ginseng.

## Discussion

This study evaluated the ameliorating effect of a seven-day ginseng treatment on the acute EO. Ginseng decreased the weight gain of the prostate and improved the percentage of normal sperm morphology, total sperm motility and sperm progressive motility in the animals induced by EO. The reduction of apoptotic indexes in the testis of ginseng treated EO rats was accompanied with an increase in both Johnsen’s and Miller’s criteria. Ginseng accepted as a modulator for body weight and sex organs weight. Although it has been shown that the saponin fraction of ginseng alleviates body weight loss in animals exposed to dioxin (24). In this study, a significant weight loss in EO animals in a seven-day treatment was not fully compensated by ginseng administration. Contrary to previous report (48), we observed no significant testicular weight loss in neither EO group nor KRG groups. Similarly, Hwang et al. showed that KRG did not affect testicular weight in aged rats (49). In our study, the relative weight of prostate and seminal vesicles increased in the EO group. Ginseng decreased prostate weight gain in EO animals. The putative role of ginseng in reducing prostate weight has also been reported by Fahim et al. (45) in intact animals. Since epididymitis or prostatic tissue inflammation depends on bacterial and nonbacterial processes (11, 50, 51), more research is needed to elucidate the probable antiinflammatory mechanism of ginseng with regard to the weight gain of accessory sex organs in acute EO.

Previous clinical and experimental studies have outlined the deleterious effects of pathogenic *E-coli* on spermatozoa parameters (48, 52, 53). Furthermore, different studies have shown the deleterious effects of experimental bacterial EO in rats such as morphological alterations, decreased motility and count of retrieved spermatozoa from the caudal part of epididymis (48, 54). *E-coli* have been shown to affect sperm morphology and motility parameters through different mechanisms. They can adhere to sperm cells and impair cellular integrity (52). Sperm immobilization factor (SIF) isolated from E-coli can also induce receptor-dependent immobilization of sperm (55). The presence of leukocytes in infected males can trigger these bacteria to produce reactive oxygen species (ROS) which can, in turn, serve as an intermediate in subsequent peroxidation of lipids in the sperm membrane (54). Previously, it has been shown that KRG increased the number mature sperm cells, the motility and the morphology of epididymal sperm under different experimental conditions (24). Probably, the protective effect of KRG on the sperm parameters may attribute to its antioxidant effects, ROS scavenging, and enhancement of the anti-oxidative defense system by attenuating free radical-induced damage caused by UPEC (28, 56).

In the present study, UPEC resulted in impairment of spermatogenesis through a reduction in Miller’s and Johnsen’s scores, and an increase in the apoptotic indices. It has been well-defined that inoculation of *E-coli* into the vas deferens, as a recognizable acute EO model, induces degeneration of germinal epithelial cells, tubular atrophy, moderate inflammation, mild interstitial fibrosis, subsequent testicular damage and DNA breakage of testicular germ cells in the seminiferous epithelium (48, 52-54). Oxidative stress and subsequent apoptosis of spermatozoa by UPEC infection is due to both invasion of germ cells by bacteria and the damage of other somatic cells or whole testicular milieu after bacteria elimination (42, 57). Our result showed that KRG improved Miller’s and Johnsen’s scores and apoptotic indices. The protective and anti- apoptotic effects of panax ginseng on the testicular germ cells have previously been reported in rats (21, 24) and rabbit testes (25) under different experimental conditions.

Apart from pivotal role of ginseng on spermatogenesis through increasing cAMP-responsive element modulator, mRNA and protein expression, its effect as an antioxidant on oxidative DNA and protein damages is attributed to its ability to elevate the levels of enzymatic and non-enzymatic antioxidants (43, 55-57) to reduce CYP1A1-mRNA in rat testes (17, 21) and to inhibit release of cytochrome C from mitochondria (19). Despite pro-inflammatory cytokines (IL-1α, IL-6 and TNF-α) synthesis in the infected epididymis and testis post UPEC incubation, immune response is ineffective against its persistent presence, and the inflammatory cytokines are also unable to release due to presence of virulence factors such as alpha-hemolysin (HlyA) (57). Similarly, our data showed that the lack of fluctuation levels of TNF-α seven days post-*E-coli* inoculation is most likely attributed to active suppression of the responsible signaling cascade in cytokine production in the testis (37). Since developing subsequent chronic inflammation is regulated by effective activation of other signaling pathways rather than viable bacteria (11), KRG may be postulated as a beneficial treatment due to its anti-inflammatory and immune-enhancer properties (38, 39). Nevertheless, other pro-inflammatory cytokines should be evaluated.

## Conclusion

Our results indicated that KRG ameliorated the devastating effects of EO on sperm retrieved from either epididymis or testicle in rats. Consequently, KRG with anti-oxidative, anti-microbial, and antiapoptotic effects could probably be a beneficial adjuvant along with antibiotic treatment. Thus, we have begun a new project on the effects of KRG and antibiotics on EO.
